# Parental Instruction

**Published:** 1914-05

**Authors:** 


### Parental Instruction.

At a recent educational conference held in Atlanta, in discussing the sex question, great stress was laid on the point that all such instructions should be given in the home. This would be good advice if the parents of the child were suitable persons to assume this responsibility. It is a lamentable fact that this is true in a very small per cent, and to limit this information to the parents would mean that the vast majority of children would grow up unprepared for the struggle of the adolescent period.

In requiring this education in the schools presupposes proper method of instruction. Nothing could be fraught with greater calamity than promiscuous half-hearted attempts. Education that excites the curiosity is dangerous. There must be a careful unfolding of the mind towards the mysteries of life—a gradual awakening, step by step, in a logical sequence. The child then grasps the idea naturally and without undue stimulation of the sex impulse.

It stands to reason that a properly trained instructor could convey knowledge better and far more safely than the average parents of today, who seem so out of tune with the sympathies of their children. To be sure, the enlightened mother who is en rapport with her child is the ideal person to impart this knowledge. And they will do so regardless of any other considerations. What we need is more such mothers, but that will not be possible until they themselves have been properly instructed and, I might say, reconstructed. Until then, and to meet the demands of the masses, let us have specially prepared teachers trained in the normal schools, for this the most important work in making our education successful.

